# Among once-daily regimens, single tablet regimens (STRs) are associated with better adherence

**DOI:** 10.7448/IAS.15.6.18101

**Published:** 2012-11-11

**Authors:** R Murri, S Fregosi, P Bortolussi, S Marcotullio, F Maggiolo

**Affiliations:** 1 Catholic University of Rome, Infectious Diseases, Rome, Italy; 2 GfK Eurisko Italy, Milan, Italy; 3 Nadir Onlus Foundation, Rome, Italy; 4 Ospedali Riuniti, Infectious Diseases, Unit of Antiviral Therapy, Bergamo, Italy

## Abstract

Previous published evidences showed that taking HAART once-daily (OD) is associated to better adherence when compared to BID or TID regimens. However, no further studies investigated whether, among OD regimens, adherence levels can be differently influenced. Aim of the study was to evaluate levels of self-reported adherence in HIV+ people according to type of HAART dosing (STR, OD with more than one pill or BID). To limit reporting biases, the study was performed in five different non-clinic settings covering North and Central Italy. A total of 230 patients on stable HAART were asked to complete a semi-structured, anonymous questionnaire reporting their attitude toward HAART, their adherence and the acceptability of their regimen. Self-perception of adherence was also investigated with a single item for comparison with real adherence behavior. Most of the subjects were males (66%) with a mean age of 46 years, with higher education level (72%) and a long history of HIV infection (mean 13.6 years). 17% of patients were on a first-line regimen. 21% reported to miss at least one dose during the past week (STR: 6%; OD >1 pill 23% and BID 21%; p<0.05). People taking STR and BID tend to report less discontinuations (all the drug of the day for at least 3 times in a month) compared to OD>1 pill (6 and 4% vs 11%). People taking therapies other than HAART reported similar adherence levels of people taking only HAART, even when stratified for dosing groups. Even people judging their adherence as ‘optimal’ or ‘very good’, 10 and 17% respectively, reported having missed a dose during the last week. At stepwise regression model, optimal adherence was correlated to being male (OR: 2.38; 95% CI: 1.19–4.74), younger (OR: 3.04; 95% CI: 1.01–9.13) and with a shorter HIV infection (OR: 3.58; 95% CI: 1.04–12.38). People taking simpler once-daily STR tend to report better adherence than people taking OD>1 pill or BID. Perception of optimal adherence is largely variable among HIV-infected people taking HAART, although only a minority of subjects showing less than perfect adherence do judge their behavior as ‘optimal’.

